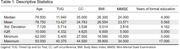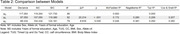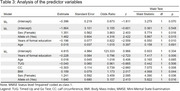# Impact of sarcopenia, frailty and apolipoprotein E ε4 allele on cognitive decline in older adults in an Outpatient Memory Clinic

**DOI:** 10.1002/alz70860_105130

**Published:** 2025-12-23

**Authors:** João Vitor da Silva Viana, Joice Coutinho de Alvarenga, Aline Siqueira de Souza, Carolina Andrade Koehne, Julia Cardoso Costa, Júlia de Almeida Barreto, Giovanna Correia Pereira Moro, Gabriela Tomé Oliveira Engelmann, Marcelle Ferreira Saldanha, Prisicila Lima Tavares, Marco Aurélio Romano‐Silva, Jonas Jardim de Paula, Maria Aparecida Camargos Bicalho, Bernardo de Mattos Viana

**Affiliations:** ^1^ Cog‐Aging Research Group, Belo Horizonte, Minas Gerais, Brazil; ^2^ Undergraduate Medicine, Faculty of Medicine, Universidade Federal de Minas Gerais (UFMG), Belo Horizonte, Minas Gerais, Brazil; ^3^ Cog‐Aging Research Group, Universidade Federal de Minas Gerais (UFMG), Belo Horizonte, Minas Gerais, Brazil; ^4^ Molecular Medicine Postgraduate Program, School of Medicine, Universidade Federal de Minas Gerais (UFMG), Belo Horizonte, Minas Gerais, Brazil; ^5^ Sciences Applied to Adult Health Postgraduate Program, School of Medicine, Universidade Federal de Minas Gerais (UFMG), Belo Horizonte, Minas Gerais, Brazil; ^6^ Older Adult's Psychiatry and Psychology Extension Program (PROEPSI), School of Medicine, Universidade Federal de Minas Gerais (UFMG), Belo Horizonte, Minas Gerais, Brazil; ^7^ Neurotec R National Institute of Science and Technology (INCT‐Neurotec R), Faculty of Medicine, Universidade Federal de Minas Gerais (UFMG), Belo Horizonte, Minas Gerais, Brazil; ^8^ Geriatrics and Gerontology Center Clinical Hospital of University of Minas Gerais, Belo Horizonte, Minas Gerais, Brazil; ^9^ Department of Psychiatry, School of Medicine, Federal University of Minas Gerais, Belo Horizonte, Minas Gerais, Brazil; ^10^ Department of Internal Medicine, School of Medicine, Federal University of Minas gerais, Belo Horizonte, Minas Gerais, Brazil

## Abstract

**Background:**

Sarcopenia is an age‐related process characterized by the loss of muscle mass and function, being a frailty component that affects the quality of life. It has been described as a risk factor for Alzheimer's disease dementia (ADD), and the coexistence of sarcopenia and ADD is associated with worse cognitive function and poor prognosis.

**Method:**

This is a cross‐sectional study, using data from the Cog‐Aging Cohort Study, including 92 participants assessed in 2023 and 2024. The Mini‐Mental State Examination (MMSE) score cut‐off was 19/20 for illiterate participants and 23/24 for literate ones, and was used to determine cognitive impairment. We aimed to evaluate the effects of age, sex, presence of the apolipoprotein E (APOE) ε4 allele, and educational level on predicting MMSE status in a Logistic Regression, with a significance level of 5%. We included Body Mass Index (BMI), Timed Up and Go Test (TUG), and calf circumference (CC) as sarcopenia and frailty variables in the model.

**Result:**

The mean age was 78.7 years (SD±7.1); median 4 years (IQR=5) of formal education, 64.1% were female and 29.3% were ε4 carriers. The MMSE median score was 24 points (IQR=5.5) and mean BMI was 26.5 (SD=±5); TUG median was 11.9 (IQR=4.3) and mean CC 34.7 (SD=±3.5). The inclusion of sarcopenia and frailty variables improved the logistic regression model (*p* = 0.035, Nagelkerke's *R* ^2^ 0.381), thus raising the performance from 68.96% to 74.71% of cases, with an AUC of 0.810. The most important factors were ε4 allele with OR of 5.15 (*p* = 0.016), followed by female sex with OR 3.46 (*p* = 0.036), and BMI with OR 1.17 (*p* = 0.04). Lower CC (*p* = 0.008) and years of formal education (*p* = 0.006) were associated with impaired MMSE status. Age and TUG did not exhibit a significant association.

**Conclusion:**

Participants with the APOE ε4 allele, higher BMI, female sex, lower formal education and lower CC showed poorer cognitive performance. Sarcopenia and frailty variables improved the overall model.